# A Rare Case of Rapid Onset Thrombocytopenia Induced by Piperacillin/Tazobactam in a Liver Transplant Recipient

**DOI:** 10.7759/cureus.18000

**Published:** 2021-09-15

**Authors:** Mais Al-Sardi, Hala Ali, Fayaz Handoo, Mohammed AlJawad

**Affiliations:** 1 Internal Medicine, King Fahad Specialist Hospital, Dammam, SAU; 2 Hepatology, King Fahad Specialist Hospital, Dammam, SAU

**Keywords:** rapid, thrombocytopenia, piperacillin, tazobactam, liver transplant

## Abstract

Drug-induced thrombocytopenia is a relatively common clinical condition. However, acute thrombocytopenia after initiation of piperacillin/tazobactam is rare, with only a few cases reported in the literature. The mechanism by which it happens is still unclear but it is thought to be immune-mediated. We present the first case of rapid-onset thrombocytopenia induced by piperacillin/tazobactam in a liver transplant recipient. Our patient had previous exposure to the antibiotic, and thrombocytopenia was treated by merely stopping the culprit antibiotic (piperacillin/tazobactam). The patient had a successful challenge with cefepime afterward despite possible cross-reactivity, making this the second case report of successful re-challenging with cefepime.

## Introduction

Drug-induced thrombocytopenia is relatively a common clinical condition. Several antimicrobial agents have been implicated to cause thrombocytopenia. Piperacillin/tazobactam is one of the commonly used broad-spectrum antibiotics in clinical practice. It has been reported as one of the infrequent causes of drug-induced thrombocytopenia with prolonged use [1]. However, immediate thrombocytopenia after starting piperacillin/tazobactam is rare and only a few cases were reported in the literature. We present the first case of rapid-onset thrombocytopenia induced by piperacillin/tazobactam in a liver transplant recipient and it is the second report of successful challenging with cefepime despite possible cross-reactivity.

## Case presentation

**Figure 1:**
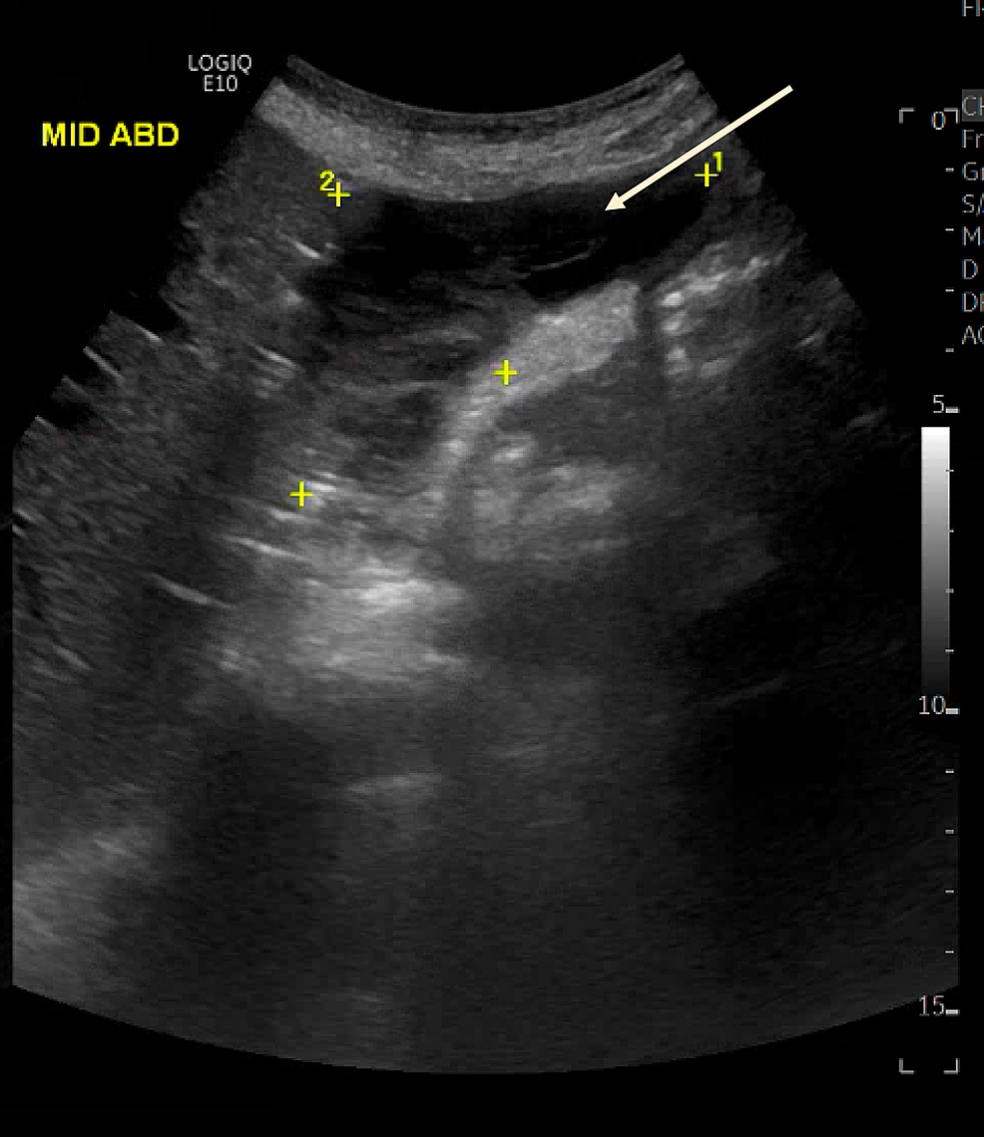
Ultrasound showing peri-hepatic fluid collection

Our patient is a 53-year-old lady known case of primary biliary cholangitis who underwent living donor liver transplantation for decompensated cirrhosis two months prior to her presentation. She was admitted to the hospital when she developed new-onset jaundice and elevated liver transaminases. She was complaining of pruritus and dark urine for five days, but there was no fever, nausea, vomiting, abdominal pain, or change in bowel habits. On examination, she was afebrile, hemodynamically stable, and her abdomen was soft and lax with no tenderness or organomegaly. Her laboratory workup showed a white blood cell (WBC) count of 10*10^9^ g/L, hemoglobin of 10.9 g/dL, platelet level of 422*10^9^ g/L, total bilirubin level of 30 µmol/L, direct bilirubin of 22 µmol/L, alkaline phosphatase of 552 unit/L, aspartate transaminase of 122 unit/L, and an alanine transaminase level of 201 unit/L. Ultrasound abdomen showed a peri-hepatic fluid collection of 140 mL indicating a possible bile leak as seen in Figure 1.

Ultrasound-guided aspiration of the perihepatic fluid collection was done and analysis of the fluid showed turbid fluid with a WBC count of 850 cells/µL and 70 neutrophils. So, the patient was started on piperacillin/tazobactam 4.5 g every six hours and the peri-hepatic fluid collection was drained. Her repeated labs the next day showed a drop in her platelet count from 425*10^9^/L to 2*10^9^/L. The blood sample was rechecked confirming the result. Blood work revealed a WBC count of 13*10^9^ g/L and her hemoglobin was stable at 10 g/dL. On examination, the patient was noticed to have bruises but no overt bleeding, her abdomen was soft and lax. Piperacillin/tazobactam was stopped the next day after labs were done. The patient had already received four doses. Her platelet count picked up the next day to 139 *10^9^/L and then normalized three days after stopping the antibiotic. During that time, the patient was on her immunosuppressive medications; tacrolimus 5 mg twice daily and prednisone 40 mg once daily (increased during admission from 7.5 mg daily as there was a suspicion of acute rejection).

Eleven days later, the patient developed leukocytosis with a WBC count of 22*10^9^ g/L. She had no fever with stable vital signs. Ultrasound revealed recurrence of peri-hepatic fluid collection. So, cultures of the perihepatic fluid collection were sent and she was started empirically on piperacillin/tazobactam 4.5 g every six hours, and a pigtail catheter was inserted to drain the collection. Her labs the next day showed a significant drop in her platelet level from 384 to 4*10^9^/L. The blood sample was rechecked confirming the same result. The patient had bleeding at the site of pigtail catheter insertion controlled with pressure dressing, but no other site of bleeding and no bruises or purpura. A peripheral blood film showed marked thrombocytopenia with no clumps or schistocytes. Her hemoglobin level was 9.4 g/dL. Her other labs showed a normal D-dimer level (0.5 mg/L), a negative direct Coomb’s test, reticulocyte count of 2.6, fibrinogen and haptoglobin levels were normal, LDH was 183 U/L and INR level was 0.9. So, piperacillin/tazobactam was stopped the next day after receiving the lab results and the patient had already taken four doses and was transfused four units of platelets. One day later, her platelet level improved to 27 and then gradually increased and normalized after three days. Then, the patient was challenged with cefepime after stopping piperacillin/tazobactam, which was well tolerated with no effect on platelet count.

The patient had previous exposure to piperacillin/tazobactam four months prior to this hospitalization as she received it for three days as a treatment for hospital-acquired pneumonia then antibiotics were de-escalated. At that time, there was a mild drop in her platelet count from a baseline of 257 to a nadir level of 144*10^9^/L.

## Discussion

Thrombocytopenia is a potentially serious side effect of medications. Similar to our case, the drug etiology could constitute a diagnostic challenge and might not be initially recognized. Thrombocytopenia could be due to decreased platelet production by myelosuppression or immune-mediated platelet destruction [2]. Drug-induced immune-mediated thrombocytopenia causes a decrease in platelet count more rapidly than myelosuppression. It has been reported to be as early as 2.5 hours post-re-exposure [3]. It has been suggested that piperacillin/tazobactam may induce antibody production after covalently binding to a platelet membrane protein [4]. Another possibility that has been proposed is via receiving piperacillin/tazobactam-specific antibodies from the donor. However, this is less likely since there was no evidence of graft-versus-host disease [5]. In our case, the chronological order of starting piperacillin/tazobactam, the degree and duration of platelet drop, and the improvement of the platelet count after stopping the antibiotic supports the provisional diagnosis of drug-induced immune-mediated thrombocytopenia. Since our patient developed thrombocytopenia rapidly after re-exposure to piperacillin/tazobactam twice, this suggests an immune sensitization. The event is further proven by the Naranjo adverse event probability scale where a calculated score of 8 makes this a probable adverse drug reaction [6]. Other causes of thrombocytopenia were considered but were unlikely as the labs showed no evidence of disseminated intravascular coagulation (DIC), her thrombocytopenia was associated with anemia and she did not have splenomegaly making ITP unlikely. Schistocytes were not present in the blood film. The patient was not on heparin and there were no other changes in her medications. Although steroids have been reported to mitigate drug-induced immune-mediated thrombocytopenia, our patient developed significant rapid thrombocytopenia despite being on prednisone 40 mg daily [3]. Starting cefepime after stopping piperacillin/tazobactam is not without risk due to possible cross-reactivity between these two antibiotics. However, platelet count was improving despite starting cefepime. To the best of our knowledge, there was only one similar reported case successfully challenged with cefepime [4].

Most reported cases of drug-induced thrombocytopenia were treated with steroids and/or intravenous immunoglobulins (IVIG) to improve the platelet count (Table 1).

**Table 1:** Literature review of drug-induced thrombocytopenia

Number of cases	Baseline platelet count	Platelet nadir	Antibody against Piperacillin	Management	Reference
2	198,000/mL, 325,000/mL	7.0 thousand/mL, 3.0 thousand/mL	Not don,e Not done	No intervention, No intervention	[1]
1	256/mm^3^	<5/ mm^3^	Not done	High dose corticosteroids	[3]
1	295,000/μL	<2,000/μL	Negative	IVIG, dexamethasone	[4]
1	216,000/mL	1,000/mL	Positive	Platelet transfusion, IVIG, prednisone	[5]
1		3x10^9^ platelets/L	Not done	Platelet transfusion, IVIG, methylprednisolone	[7]
1	193,000/mL	10,000/µL	Positive	Corticosteroids	[8]
1	317 x 10^3^/mm^3^ to	7 x 10^3^/mm^3^	Not done	Platelets	[9]
2	230 × 10^9^/L, 274 × 10^9^/L	3 × 10^9^/L, 7 × 10^9^/L	Not done, Not done	IVIG, Methylprednisolone IVIG, Dexamethasone	[10]
1		2×10^9^/L	Not done	Platelet transfusion, IVIG	[11]
1	200,000/μL	6,000/μL	Positive	Platelet transfusion, IVIG	[12]
1	99,000/mm^3^	19,000/mm^3^	Not done	Hemodialysis (patient had end-stage renal disease)	[13]
1	377,000/µL	18,000/µL		Platelet transfusion	[14]
1		3 x 10^3^/mm^3^	Not done	Hemodialysis (patient had end-stage renal disease)	[15]

Our patient’s platelet count improved spontaneously after stopping the probable offending agent (piperacillin/tazobactam), keeping in mind she was already on prednisone as mentioned. Interestingly, one of the reported cases had no improvement in platelet level after administration of steroids and IVIG and continuing piperacillin/tazobactam. That might indicate that the mainstay of management is stopping the culprit antibiotic and there is possibly no need for further management as it was seen in our case too [7]. As there were few other cases reported to have spontaneous improvement after stopping piperacillin/tazobactam [1,9,14].

## Conclusions

In conclusion, piperacillin/tazobactam could cause rapid onset immune-mediated drug-induced thrombocytopenia. This report suggests that caution and vigilance should be exercised when using this antibiotic since the delay in making the diagnosis might cause severe thrombocytopenia that potentially could have severe and fatal sequelae. The mainstay of treatment is early recognition and discontinuation of the offending agent.
